# Future Prospective and Current Trend of Biomaterials and Growth Factor Used for Maxillofacial Hard and Soft Tissue Reconstruction

**DOI:** 10.1155/2018/3249653

**Published:** 2018-11-18

**Authors:** Marco Cicciù, Tolga Tozum, Pablo Galindo Moreno, Luigi Laino

**Affiliations:** ^1^Department of Biomedical and Dental Sciences and Morphological and Functional Imaging, Messina University, 98100 Messina, Italy; ^2^Department of Periodontics, College of Dentistry, University of Illinois at Chicago, Chicago, Illinois, USA; ^3^Oral Surgery and Implant Dentistry Department, School of Dentistry, University of Granada, Granada, Spain; ^4^Multidisciplinary Department of Medical-Surgical and Odontostomatological Specialties, University of Campania “Luigi Vanvitelli”, 80121 Naples, Italy

In the last few years, several brands of biomaterials, such as natural and artificial polymers, ceramics, and metals, were developed in the clinical application. In the maxillofacial area, different types of materials have been applied for bone regeneration and for replacing autologous bone graft [1–3].

Tissue engineering and stem cell carrier materials usually develop tissue-biomaterial interactions that suitably recapitulate a tissue or organ and integrate well with surrounding tissues, which can achieve the desired results in human patients and greatly reduce postoperative discomfort. The maxillofacial surgery application of growth factor carriers and bone substitute has considerably broadened within the last decades. The use of bioengineering materials needs to move towards new dental treatment concepts based on being safe and predictable for daily practice application. The shape and microscopical structure of novel materials should be able to give clinicians the possibility of safely obtaining the new tissue in the place of the pathological, removed hard and soft tissue [4, 5].

It was a pleasure to invite contributors and researchers to submit original research articles as well as review articles to state and extend the field of biomaterial and maxillofacial reconstruction techniques.

The topic is pretty wide and this special issue may be directed also to anatomy, histology, biology, and bioengineering researchers in order to have a current understanding of the state of the art in all the possible applications. Topics include new biomaterials and techniques of regenerative and reconstructive surgery; new scaffold used for growth factor carriers; new therapies for large hard and soft tissue reconstruction in the maxillofacial area; new bone graft strategies for reducing patient postoperative discomfort; new tissue engineering devices for having safe, predictable, and long-term results in their clinical application.

A number of fifteen papers were submitted for this special issue. Our distinguished reviewers from respective research fields underlined the field to eight papers, which were finally accepted. The following is a short summary of the outcomes of each of these manuscripts.

D. Baldi et al. evaluated the correlation between insertion torque (IT) and implant stability quotient (ISQ) in tapered implants with knife-edge threads. The authors concluded that the strength of the association between IT and ISQ value was significant for both the entire sample and the medium torque group, while it was not significant in low and high torque groups. For the investigated implant, ISQ and IT showed a positive correlation up to values around 50 N/cm: higher torques subject the bone-implant system to unnecessary biological and mechanical stress without additional benefits in terms of implant stability.

Y.-T. Wang et al. analyzed the structural optimization of anatomical thin titanium mesh (ATTM) plate and optimal designed ATTM plate fabricated using additive manufacturing (AM) to verify its stabilization under fatigue testing. This study concluded that the optimal designed ATTM plate with enough strength to resist the bending effect can be obtained by combining FE and Taguchi analyses. The optimal designed ATTM plate with patient-matched facial contour fabricated using AM provides superior stabilization for ZMC comminuted fractured bone segments.

J. Li et al. investigated effect of aimed-control design on mass transfer and tissue regeneration of porous implant with regular unit cell. Two shapes of unit cells (Octet truss and Rhombic 21 dodecahedron) were selected, which have similar symmetrical structure and are commonly used in practice. This study confirmed that porous implant with different unit cell shows different performances of mass transfer and tissue regeneration, and that unit cell shape and strut size play vital roles in the control design. These findings could facilitate the quantitative assessment and optimization of the porous implant.

O. B. Agrali et al. evaluated the regenerative capacity of HA matrix in rat calvarial bone defects compared with those of different combinations of resorbable collagen membrane (M) and bovine derived xenograft (G). The authors concluded that HA matrix, used alone or in combination with G and M, did not contribute significantly to bone regeneration in rat calvarial bone defects.

R. L. Giudice et al. evaluated if a steam sterilization could provide a medical grade sterilization of the blocks and if bone microstructure and collagen structures change after different steam sterilization protocols provided by mainstream autoclave. Data show that autoclave steam sterilization could be reliable to obtain sterilization of equine bone blocks.

R. Rullo et al. assessed, with clinical, radiographic, and histological evaluations, the efficacy of piezoelectric devices compared to traditional rotating instruments in the bone harvesting in patients with history of cleft. The authors concluded that the application of the piezoelectric items in bone harvesting allows a slight improvement in the final volume. This supports a faster integration into the receiving site. The use of piezoelectric device in patients with history of orofacial cleft that needed bone graft represents a method to be taken into consideration because it has interesting advantages.

T. Lombardi et al. researched, with three-dimensional analysis, the effectiveness of alveolar ridge preservation (ARP) after maxillary molar extraction in reducing alveolar bone resorption and maxillary sinus pneumatization when compared to unassisted socket healing. The authors suggested that ARP performed after maxillary molar extraction may reduce the entity of sinus pneumatization and alveolar bone resorption, compared to unassisted socket healing. This technique could decrease the necessity of advanced regenerative procedures prior to dental implant placement in posterior maxilla.

N. Mazzone et al. reported their experience using a synthetic bone substitute in combination with Platelet Rich Fibrin (PRF). This technique was applied in different zones of the maxillomandibular district. The procedure showed satisfying bone regeneration without important complications
